# Acceleration of Autoimmunity by Organochlorine Pesticides in (NZB × NZW)F_1_ Mice

**DOI:** 10.1289/ehp.7347

**Published:** 2004-12-02

**Authors:** Eric S. Sobel, John Gianini, Edward J. Butfiloski, Byron P. Croker, Joel Schiffenbauer, Stephen M. Roberts

**Affiliations:** ^1^Department of Medicine, and; ^2^Department of Pathology, Immunology, and Laboratory Medicine, University of Florida College of Medicine, Gainesville, Florida, USA; ^3^Northern Florida/Southern Georgia Veterans Health System and Laboratory Medicine Service, Gainesville, Florida, USA; ^4^Department of Physiological Sciences, J. Hillis Miller Health Science Center, University of Florida, Gainesville, Florida, USA

**Keywords:** autoimmunity, chlordecone, DDT, estrogenicity, glomerulonephritis, kepone, methoxychlor, organochlorine pesticides, systemic lupus erythematosus

## Abstract

Systemic lupus erythematosus (SLE) is an autoimmune disorder that affects women more frequently than men. In the (NZB × NZW)F_1_ mouse, a murine SLE model, the presence or absence of estrogen markedly influences the rate of progression of disease. Three organochlorine pesticides with estrogenic effects were administered chronically to ovariectomized female (NZB × NZW)F_1_ mice, and we measured the time to development of renal disease, the principal clinical manifestation of lupus in this model. Treatment with chlordecone, methoxychlor, or *o*,*p*′-dichlorodiphenyl-trichloroethane (*o*,*p*′-DDT) significantly decreased the time to onset of renal impairment, as did treatment with 17β-estradiol used as a positive control. In an expanded study of chlordecone, we found a dose-related early appearance of elevated anti–double-strand DNA autoantibody titers that corresponded with subsequent development of glomerulonephritis. Immunohistofluorescence confirmed early deposition of immune complexes in kidneys of mice treated with chlordecone. These observations are consistent with an effect of these organochlorine pesticides to accelerate the natural course of SLE in the (NZB × NZW)F_1_ mouse. Although we originally hypothesized that the effect on progression of autoimmunity was due to estrogenic properties of the pesticides, autoimmune effects and estrogenicity, assessed through measurement of uterine hypertrophy, were not well correlated. This may indicate that uterine hypertrophy is a poor indicator of comparative estrogenic effects of organochlorine pesticides on the immune system, or that the pesticides are influencing autoimmunity through a mode of action unrelated to their estrogenicity.

Systemic lupus erythematosus (SLE) is a chronic, systemic autoimmune disorder characterized by exacerbations and remissions of varying intensity and duration. Its clinical manifestations are almost invariably accompanied by the presence of autoantibodies directed at a wide array of self-components, including cell-surface structures (surface proteins and phospholipids on lymphocytes) and intracellular molecules (DNA, histones, and RNA) (von Muhlen and Tan 1995). Among its many manifestations, the presence of renal disease in the form of glomerulonephritis is used as a major predictor of morbidity and mortality (Ansell et al. 1996).

Current evidence suggests that a combination of factors plays a role in the development of autoimmunity in SLE, including genetic, environmental, hormonal, and viral influences (D’Cruz 2000; Roubinian et al. 1978). Although SLE can be seen in either sex at any age, females are at a much greater risk than are males, with the highest incidence occurring in women in their childbearing years (Lahita 1996). Although the precise factors responsible for the increased incidence of disease in women remain to be clarified, several lines of evidence suggest that sex hormones may be a predisposing factor (Cutolo et al. 1995; Rood et al. 1998; Sanchez-Guerrero et al. 1997).

An influence of estrogen status on the development of SLE is clearly demonstrated in murine SLE models, such as the (NZB × NZW)F_1_ mouse. Although most (NZB × NZW)F_1_ mice develop SLE spontaneously and experience premature mortality, females develop more severe disease and succumb at an earlier age than do males (Roubinian et al. 1978). Androgen treatment of females decreases autoantibody levels, diminishes renal disease, and improves survival (Roubinian et al. 1979). In males, castration or administration of estrogen accelerates development of SLE.

Several environmental contaminants have been found to produce estrogen-like effects, including some of the organochlorine pesticides (OCPs) (e.g., Andersen et al. 2002; Soto et al. 1994). Given the influence of estrogen on SLE in experimental models, we hypothesized that OCPs with estrogenic effects would similarly accelerate lupus development. As an initial test of this hypothesis, three different OCPs were tested for effects on the time course of development of lupus in female (NZB × NZW)F_1_ mice, *o*,*p*′-dichlorodiphenyltrichloroethane (*o*,*p*′-DDT), methoxychlor, and chlordecone. Each of these pesticides had been shown previously to possess estrogenic activity *in vivo* (e.g., Cummings 1997; Das-Sanjoy et al. 1998; Flaws et al. 1997; Morozova et al. 1997). Mice were ovariectomized to reduce the potentially confounding effects of cycling endogenous estrogen and were treated chronically with one of the pesticides or with 17β-estradiol as a positive control. Time to development of clinical SLE, manifested as terminal immune complex glomerulonephritis, was measured. In these experiments, all of the OCPs caused acceleration of time to development of SLE, with chlordecone producing the greatest response. More detailed, follow-up experiments were conducted with chlordecone to examine dose–response relationships and verify estrogenic effects. The results of these experiments, reported here, indicate an effect of the OCPs to speed progression of autoimmunity in (NZB × NZW)F_1_ mice.

## Materials and Methods

### Mice.

Female (NZB × NZW)F_1_ mice, 6–8 weeks of age, were obtained from the Jackson Laboratory (Bar Harbor, ME). Mice were housed in temperature-, light-, and humidity-controlled animal quarters under specific-pathogen-free conditions. Polycarbonate cages with ground corncob bedding were used, and mice had free access to food and water for the duration of the experiment. All procedures were approved by the institutional animal care and use committee of the University of Florida.

### Ovariectomy.

All animals were subjected to ovariectomy or a sham-operation approximately 1 week after they were received. Surgery was performed under anesthesia with a mixture of ketamine (133 mg/kg) and xylazine (13 mg/kg) administered intraperitoneally. All procedures were identical for ovariectomized and sham-surgery mice, except that in the latter case, ovaries were exposed but not ligated and removed.

### Test materials and treatments.

We purchased chlordecone (described as 99.2% pure) from Crescent Chemical (Islandia, NY). methoxychlor (95%) from Sigma Chemical Co. (St. Louis, MO), and *o*,*p*′-DDT (98%) from Chem Service (Westchester, PA). We confirmed the identity of each material as supplied by gas chromatography–mass spectrometry. Each pesticide was formulated in specified amounts into sustained-release pellets (60-day release) by Innovative Research of America (Sarasota, FL). Control pellets (matrix only), and pellets containing 17β-estradiol were also obtained from this source. One week after ovariectomy, mice were implanted subcutaneously with an OCP, 17β-estradiol, or control pellet. For implantation, mice were lightly anesthetized with methoxyflurane, and the pellet was placed beneath the skin dorsally above the shoulders. Implants were replaced every 60 days for the duration of the experiment.

### Evaluation of renal function.

Once the pellets were implanted, we systematically evaluated the course of the disease. The appearance of renal disease was detected through monthly measurement of urine protein and blood urea nitrogen (BUN). Urine was collected from spontaneous expression during manual restraint. Proteinuria was measured by dipstick method using Albustix (Bayer Corp., Elkhart, IN). Blood samples were obtained from tail nicks, and BUN was measured using Azostix (Bayer Corp.). Body weights were also recorded monthly. If animals showed weight loss of ≥10% or worsening renal function by either BUN or proteinuria, they were reassessed every 2 weeks until termination. Mice were euthanized when the BUN reached or exceeded 50 mg/dL and/or proteinuria reached or exceeded 2,000 mg/dL. In the initial study of three OCPs, surviving mice were euthanized 8 months after the start of treatment. In the second study focusing on chlordecone, the experiment was ended and surviving mice euthanized after 7 months of treatment.

### Histology.

At defined time points, mice from each group were euthanized, and their kidneys were preserved in 10% buffered formalin. Tissue samples were processed routinely, sectioned, mounted on glass slides, and stained with hematoxylin and eosin or periodic acid Schiff (PAS) for light microscopic examination. All kidney sections were viewed and scored by a pathologist in a blinded examination. The glomeruli were categorized using a modified World Health Organization classification (Churg et al. 1995) as having no damage, mesangiopathic damage, or proliferative damage. In addition to the severity of the glomerular damage, the amount of renal damage was quantified using the following scale: 1+, 1–9% of glomeruli affected; 2+, 10–24% of glomeruli affected; 3+,(25–49% of glomeruli affected; and 4+, ≥50% of glomeruli affected.

### Autoantibody titers.

We measured auto-antibody [IgG anti–double-strand DNA (anti-dsDNA)] titers in serum in some treatment groups using indirect ELISA (enzyme-linked immunosorbent assay). Immulon 2 microtiter plates (Dynatech Laboratories, Inc., Chantilly, VA) were coated overnight at 4°C with a 1:10 (vol/vol) dilution of poly-l-lysine at 100 μL/well. Between all steps, microplates were washed three times with borate-buffered saline (25 mM Na_2_B_4_O_7_, 75 mM NaCl, 100 mM H_3_BO_3_, pH 8.4) containing 0.05% Tween 20. After addition of calf thymus DNA (50 μL/well at a concentration of 20 μg/mL), the plate was blocked with 100 μL/well of 3% bovine serum albumin (BSA) in phosphate-buffered saline (PBS). Dilutions (1:200) of serum samples were prepared in PBS and incubated in the appropriate wells at room temperature for 1 hr. The secondary antibody (goat anti-mouse IgG peroxidase conjugated F_c_ gamma specific; Jackson ImmunoResearch Laboratories, West Grove, PA) was diluted to 1:5,000 (vol/vol) in PBS and added at 50 μL/well. The developing solution consisted of *o*-phenylene diamine at 0.4 mg/mL PC buffer (4.7 g/L citric acid and 13.8 g/L sodium phosphate dibasic heptahydrate, pH 5.1) with 0.01% H_2_O_2_. The substrate turnover was determined by the difference between the OD_450_ (optical density) and OD_620_ on a Molecular Devices (Sunnyvale, CA) microplate reader. The concentration of antigen-specific IgG is reported in equivalent dilution factors (EDFs) of standardized reference NZB × NZW/F_1_ sera. This is defined by the formula EDF = (dilution of a standard reference sera that gives the equivalent OD of the test serum) × 10^4^.

### Immunohistofluorescence.

Immune complex deposition in glomeruli was visualized by immunohistofluorescence. Renal tissue sections 7 μm in thickness were mounted to glass slides (Fisher Scientific, Fairlawn, NJ). Blocking solution (1% BSA, 0.1% Tween, and 10% rat serum in PBS) was applied for 20 min. Then slides were incubated with 100 μL goat anti-mouse IgG–fluorescein isothiocyanate antibody (Southern Biotech, Birmingham, AL) diluted 1:40 in PBS with 1 mg/mL BSA for 30 min in a humidified chamber. Slides were then washed three times in PBS and once in distilled water. One drop of glycerin was placed on the sample, and the slide was coverslipped and examined by fluorescence microscopy.

### Uterine hypertrophy.

We conducted a separate, short-term experiment in which we evaluated the estrogenicity of the chlorinated pesticides at various doses. Ovariectomized mice were implanted with a control pellet or a pellet containing pesticide or 17β-estradiol as in the chronic experiments. After a 4-week period, the mice were euthanized, and estrogenicity of the doses was determined by measurement of wet uterine weight. Immediately after euthanasia, the uteri were excised, trimmed free of fat, pierced, and blotted to remove excess fluid. The body of the uterus was cut just above its junction with the cervix and at the junction of the uterine horns with the ligating clips. The uterus was then weighed (wet weight). To compensate for the mass of the mouse, uterine weight was expressed as a ratio to total body weight (i.e., uterine mass/body mass × 1,000).

### Statistical analysis.

We performed statistical analysis for survival using GraphPad Prism 3 software (GraphPad Software Inc., San Diego, CA). Uterine wet ratios were compared for significance using Dunnett’s procedure of one-way analysis of variance (ANOVA). Proteinuria and BUN data were tested using Dunnett’s nonparametric procedure of the Kruskal-Wallis ANOVA on ranks. The autoantibody levels were natural log-transformed before analysis in order to normalize their distribution and improve the accuracy of the analysis. After transformation, statistical significance was determined using Dunnett’s method of ANOVA. Survival data were displayed as Kaplan-Meier survival curves. We determined differences between survival curves using the log rank test. For the dose–response experiment, we also performed the log rank test for trend.

## Results

We conducted an experiment in which we assessed the effects of chronic treatment with chlordecone, *o*,*p*′-DDT, or methoxychlor on time to development of lupus in ovariectomized (NZB × NZW)F_1_ mice. The objective of this experiment was to provide an initial test of the hypothesis that estrogenic OCPs accelerate the rate of development of disease in this lupus model. Chronic doses of these pesticides producing estrogenic effects in mice were not clearly established in the literature and were therefore estimated based upon short-term studies in the literature of potential estrogenic effects *in vivo*. Two doses of each OCP were tested: 1.8 and 18 mg chlordecone, 0.9 and 9 mg *o*,*p*′-DDT, and 3 and 30 mg methoxychlor. As a positive control, we also tested 0.1 mg 17β-estradiol. These doses were the amount of chemical delivered over each 60-day interval via implanted pellets. Ovariectomized females given pellets with matrix only served as controls, and an additional comparison group of untreated, ovary-intact (sham operated) females was included in the experiment. Each treatment group contained 10 animals. Within a few weeks of the start of the experiment, mice treated with the 18-mg chlordecone pellets developed tremors and were removed from the study. Overt neurotoxicity was not observed in the other treatment groups.

**Figure 1:**
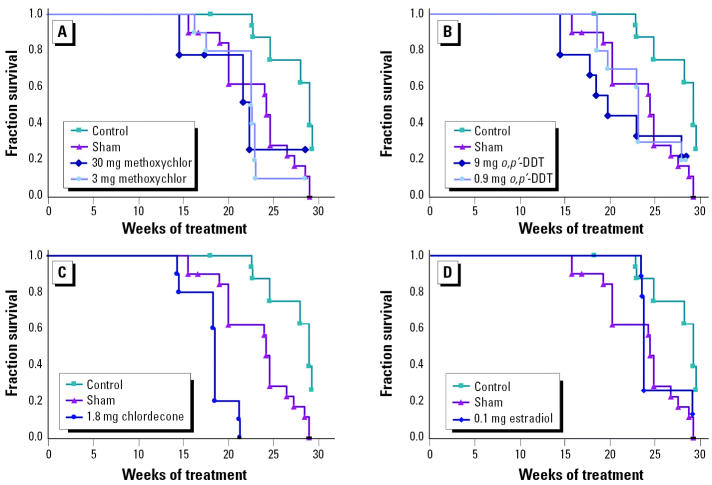
Time to development of renal disease (plotted as a survival curve) in ovariectomized (NZB × NZW)F_1_ mice implanted with pellets containing (*A*) methoxychlor, (*B*) *o*,*p*′-DDT, (*C*) chlordecone, or (*D*) 17β-estradiol; ovariectomized mice implanted with control pellets and sham-operated mice are shown for comparison (*n* = 10/group). The time to development of renal disease was significantly decreased in all OCP-treated groups compared with ovariectomized controls (*p* < 0.05)

The primary assessment end point for this experiment was the development of severe renal disease, the hallmark of lupus in this animal model. Time to development of severe renal disease for each treatment group is shown in the form of Kaplan-Meier survival curves in Figure 1. Ovariectomy extended substantially the time to development of renal disease in this mouse strain. This is evident by comparing the time courses in Figure 1 for the ovariectomized controls with the sham-operated (i.e., ovary intact) comparison group. Replacement of estrogen with 17β-estradiol (0.1 mg/pellet) in ovariectomized mice shortened the time to appearance of renal disease such that it was not significantly different from that of sham-operated mice (Figure 1D).

Treatment of ovariectomized mice with *o*,*p*′-DDT or methoxychlor significantly shortened the time to development of renal disease compared with controls (Figure 1A,B). The survival plots for *o*,*p*′-DDT–treated and methoxychlor-treated groups were essentially the same as the plot for the sham-operated comparison group, suggesting an influence on lupus development roughly equivalent to that of endogenous estrogen. There was no significant difference in survival curves between the two doses tested for *o*,*p*′-DDT and methoxychlor, although the higher *o*,*p*′-DDT doses appeared to result in more rapid development of renal disease. The time to development of renal disease in the single chlordecone-treated group completing the study was significantly shorter than both the ovariectomized controls and the untreated, sham-surgery comparison group (Figure 1C). The difference in survival was particularly striking after 22 weeks of chlordecone treatment: 100% of the ovariectomized controls, 60% of the mice in the sham-surgery group, and none of the chlordecone-treated mice survived to this time point.

**Figure 2:**
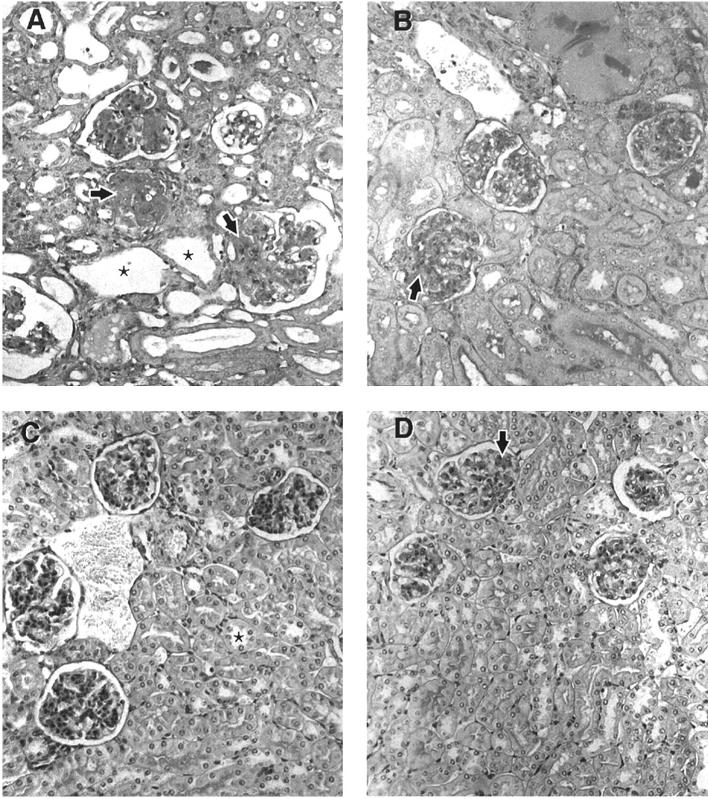
Representative kidney sections taken from (NZB × NZW)F_1_ mice after 16 weeks of treatment. (*A*) Sham-operated mouse. (*B*) Ovariectomized mouse treated with chlordecone (1.8 mg/pellet). (*C* and *D*) Two ovariectomized mice, each treated with a control pellet. (*A*) and (*B*) show enlarged, cellular, and sclerotic glomeruli (arrows) with tubular atrophy and dilation (asterisks). (*C*) and (*D*) show segmental mesangial thickening, but otherwise spared, glomeruli (arrows) with normal tubules (asterisk). Sections were stained with PAS (magnification, 400×).

Additional mice were implanted with 1.8 mg chlordecone or control pellets and euthanized after 16 weeks of treatment. Measurement of proteinuria at this time point indicated severe renal disease in the chlordecone-treated mice but not in the ovariectomized controls. Renal sections from these mice were examined by light microscopy (Figure 2). The observations correlated well with proteinuria findings and were consistent with early appearance of immune complex glomerulonephritis in chlordecone-treated mice. All of the chlordecone-treated mice had developed significant proliferative glomerulonephritis with fibrosis, the most severe form of glomerulonephritis in lupus. The control mice, in contrast, showed only mesangial involvement, an early or mild form of renal disease. One control animal had evidence of proliferative glomerulonephritis, but only approximately 10% of glomeruli were affected.

**Figure 3:**
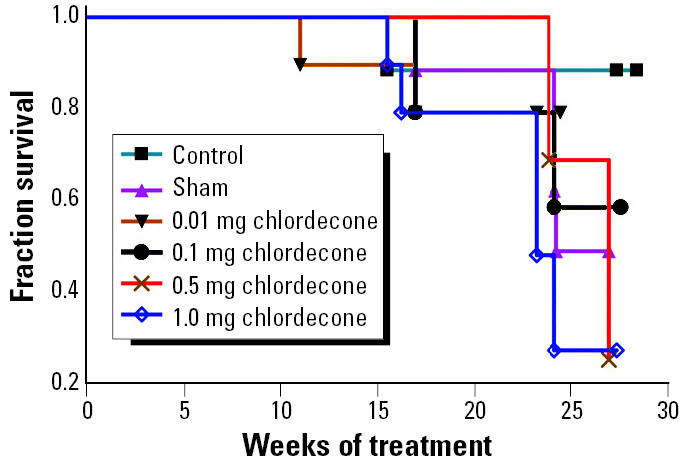
Time to development of renal disease in ovariectomized (NZB × NZW)F_1_ mice (*n* = 20 controls; *n* = 10 sham operated; *n* = 10 for each of the chlordecone-treated groups). The time to appearance of renal disease was significantly decreased in mice treated with the 1-mg chlordecone pellets (*p* < 0.05).

In view of the particularly strong response to chlordecone, we conducted a follow-up experiment in which lower doses of chlordecone were tested (0.01, 0.1, 0.5, and 1.0 mg per 60-day-release pellet). The control group (matrix-only pellets) consisted of 20 animals; all other groups consisted of 10 animals each. The time course for development of severe renal disease is shown in Figure 3. Mice treated with the 1.0 or 0.5 mg chlordecone pellets developed renal disease significantly earlier than did ovariectomized controls (*p* < 0.05). The rate of development of lupus also appeared to be enhanced in mice treated with 0.01 and 0.1 mg chlordecone pellets, although the difference did not reach statistical significance. The log rank test for trend using all three doses was statistically significant (*p* < 0.03).

**Figure 4:**
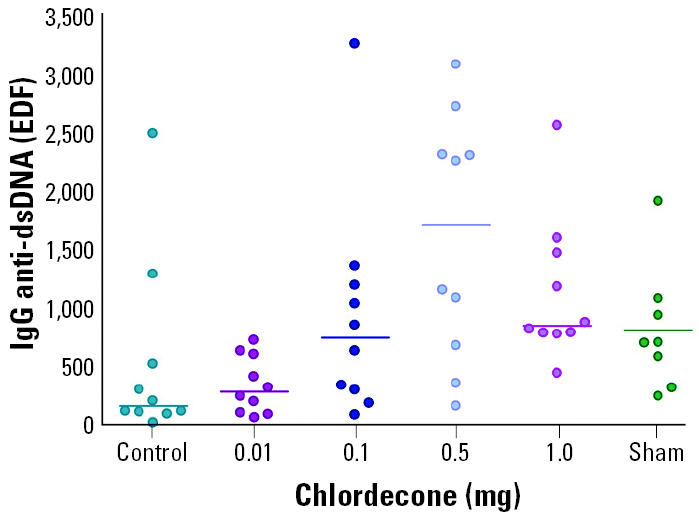
Serum autoantibody titers in ovariectomized (NZB × NZW)F_1_ mice treated 20 weeks after treatment with chlordecone. Ovariectomized mice implanted with control pellets and sham-operated mice are shown for comparison. Antibody titers from mice treated with 1 mg chlordecone pellets were significantly higher than those of ovariectomized controls (*p* < 0.01).

During the course of the follow-up experiment with chlordecone, mice were bled periodically, and the sera were tested for anti-dsDNA by ELISA. Elevated autoantibody titers appeared earlier in chlordecone-treated mice than in ovariectomized controls. The greatest difference was seen after 20 weeks of treatment, a time point in this experiment at which substantial differences in overt renal disease had not yet appeared (Figure 3). Mice treated with 1 mg chlordecone pellets had titers significantly higher than those of controls (*p* < 0.01) and essentially equivalent to those in the sham-operated comparison group (Figure 4). Autoantibody titers in mice treated with 0.1 mg chlordecone were also elevated compared with controls, but the difference was not statistically significant.

**Figure 5:**
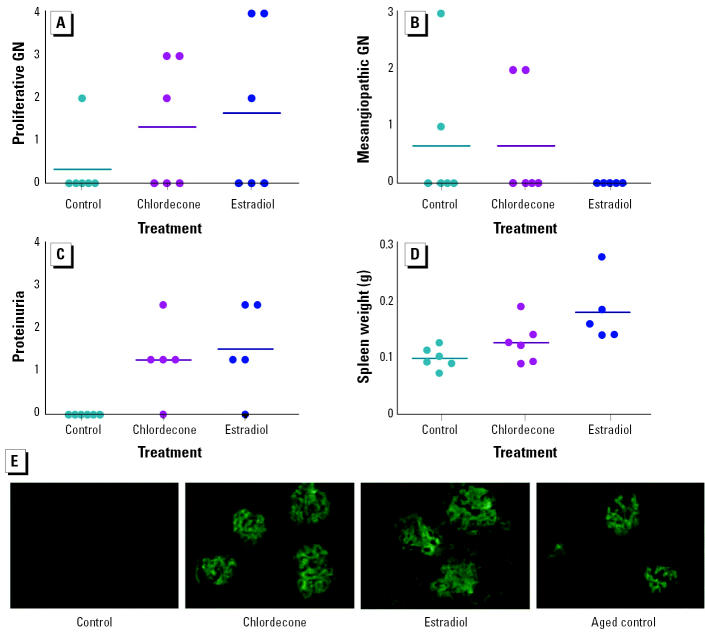
Enhanced renal disease and immune complex deposition in ovariectomized, chlordecone-treated mice after 8 weeks of treatment with control pellets or pellets containing chlordecone (1 mg/pellet) or 17β-estradiol (0.05 mg/pellet; *n* = 6/group). Frequency of appearance of proliferative glomerulonephritis (GN; *A*), mesangiopathic glomerulonephritis (*B*), and proteinuria (*C*), and spleen weight. The frequency of occurrence of proteinuria was significantly increased in both chlordecone-treated and 17β-estradiol–treated mice; spleen weight (*D*) was significantly increased by 17β-estradiol. (*E*) Immunofluorescence staining (magnification, 200×) for IgG was absent in control mice, but present in mice treated with either chlordecone or 17β-estradiol after 8 weeks of treatment; later, when renal disease developed in controls, a similar extent of immunofluorescence staining was observed.

To verify that the earlier renal failure in chlordecone-treated mice was of autoimmune origin, a separate small cohort of (NZB × NZW)F_1_ female mice was ovariectomized and individual animals were implanted with a control pellet or a pellet containing chlordecone (1 mg) or estradiol (0.05 mg). Approximately 8 weeks later, at a time when some of the mice were beginning to show proteinuria, all mice were euthanized. The spleens were weighed, and the kidneys were evaluated for histopathology and immunostained for IgG. By light microscopic examination, there was a tendency toward worsened proliferative glomerulonephritis in the chlordecone- and estradiol-treated groups (Figure 5A). In contrast, less severe mesangiopathic changes were decreased, particularly in the estradiol-treated groups (Figure 5B). In general, proliferative and mesangiopathic changes were not seen on the same specimens. Proteinuria was greater in both the chlordecone- and estradiol-treated groups (Figure 5C), and both differences were statistically significant when compared to ovariectomized controls (*p* < 0.05). Spleen weight (Figure 5D) was clearly increased in the estradiol-treated group, and this reached statistical significance (*p* < 0.05). We also observed a tendency toward increased spleen weight in the chlordecone-treated group, but it did not reach statistical significance (*p* = 0.14). These observations were consistent with previous experiments showing similar, early development of severe renal disease in chlordecone-and estradiol-treated (NZB × NZW)F_1_ mice (Figure 1). Immunohistofluorescence analysis of renal sections showed enhanced deposits of IgG in the chlordecone- and estradiol-treated groups (Figure 5E). Little or no immuno-staining was observed in the control group at this time point. As indicated by previous experiments, had the control mice been observed for longer periods, they ultimately would have developed proteinuria and renal disease. For perspective on the extent of immune complex deposition in controls at these later time points, renal sections from controls from previous experiments, taken at the time of development of renal disease, were also immunostained. As shown in Figure 5E, the immune complex deposition when renal disease appeared was similar in control, chlordecone-treated, and estradiol-treated mice. These observations offer additional support for the concept that chlordecone treatment results in earlier appearance of renal disease by accelerating the course of autoimmune disease in a manner similar to estrogen.

**Figure 6:**
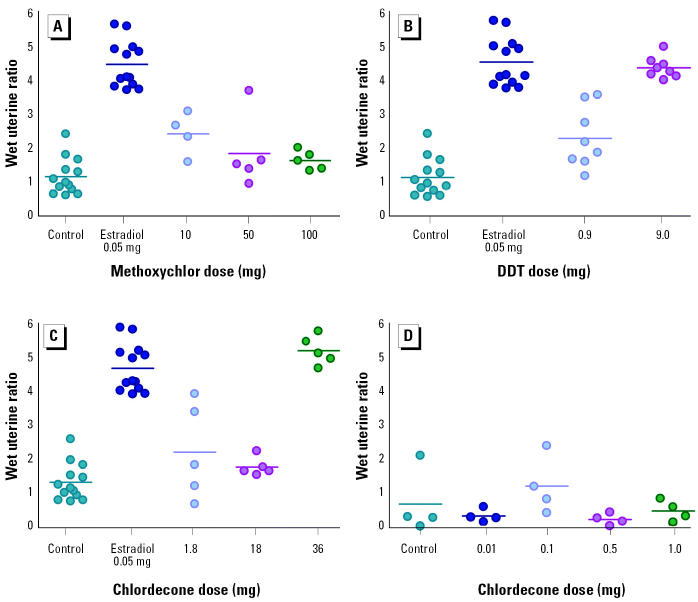
Uterine hypertrophy in ovariectomized (NZB × NZW)F_1_ mice 6 weeks after administration of pellets containing (*A*) methoxychlor, (*B*) *o*,*p*′-DDT, and (*C*) higher and (*D*) lower doses of chlordecone. Results from controls and 17β-estradiol–treated mice are reproduced in A–C to facilitate comparison with OCP-treated mice. Compared with controls, uterine weights were significantly increased in mice treated with 0.05 mg estradiol, 10 mg methoxychlor, 0.9 and 9.0 mg *o*,*p*′-DDT, and 36 mg chlordecone.

The estrogenicity of chlordecone, methoxychlor, and *o*,*p*′-DDT in doses relevant to the autoimmunity experiments was tested using the classical end point of uterine hypertrophy. (NZB × NZW)F_1_ mice were ovariectomized and subsequently implanted subcutaneously with a control pellet or a pellet containing one of the pesticides. Additional mice were treated with a pellet containing 17β-estradiol as a positive control. Six weeks after implantation, mice were euthanized, and the uterine wet weight and total body weight were measured. Chlordecone and *o*,*p*′-DDT produced uterine hypertrophy (Figure 6), although, except for the highest doses of *o*,*p*′-DDT and chlordecone tested, the effects were substantially less than those produced by estradiol (0.5 mg/pellet). Chlordecone was tested over the broadest range of doses. Chlordecone doses of ≥18 mg/pellet were required to produce significant uterine hypertrophy, despite observations in previous experiments that doses as low as 1 mg/pellet were sufficient to influence the rate of development of lupus.

## Discussion

It has long been hypothesized that environmental factors influence the onset and course of autoimmune diseases. Despite this, the number of chemicals clearly shown to influence autoimmunity is relatively small. Although a number of potential mechanisms can be postulated, they are thought to fall into three general categories (Rao and Richardson 1999). The first category is one in which the chemical alters self antigen such that it appears foreign to the immune system. This can occur when small molecules act as haptens or if exposure can cause novel cleavage fragments to which the immune system was ignorant. Heavy metals, such as mercury, may be an example of this pathway. The second category is one in which the chemical prevents the central tolerance of autoreactive T or B cells and is represented by pro-cainamide hydroxylamine. The third category involves alteration of gene expression. Hormones, such as estrogens, are thought to belong to this last category (Grimaldi et al. 2002). Because many of the OCPs have been shown to have estrogenic effects, we decided to test representative estrogenic OCPs in the well-characterized (NZB × NZW)F_1_ model of murine lupus.

The chronic administration of chlordecone, methoxychlor, or *o*,*p*′-DDT via implantable pellets significantly shortened the time to development of lupus in ovariectomized female (NZB × NZW)F_1_ mice. The most extensive experimentation was conducted with chlordecone and showed a dose-related, early appearance of elevated anti-dsDNA autoantibody titers and immune complex deposition in the kidneys. Doses of chlordecone that significantly elevated autoantibody titers at early time points also significantly reduced the time to subsequent development of renal disease, which was confirmed to be immune complex glomerulonephritis, the hallmark for clinical lupus in this murine model. Collectively, these observations indicate that chlordecone, and probably also methoxychlor and *o*,*p*′-DDT, acts by accelerating the natural course of development of lupus in these animals.

For chlordecone, the lowest dose per pellet found to produce a significant decrease in time to onset of renal disease was 0.5 mg. Over the course of the experiment, mice weighed on average about 42 g, resulting in a dosing rate per unit body weight of approximately 0.20 mg/kg/day. The next lower dose of chlordecone tested, 0.1 mg per pellet (or about 0.04 mg/kg/day), might be considered a no observable effect level (NOEL) from this experiment. However, there was evidence for an effect at this level and even at the lowest dose of chlordecone tested (0.01 mg/pellet). It is possible that with the use of larger treatment groups, effects from these lower doses might also reach statistical significance. Other effects of chlordecone, such as renal and liver toxicity, are generally associated with chronic chlordecone doses of 0.05 mg/kg/day or higher in rodents [Agency for Toxic Substances and Disease Registry (ATSDR) 1995].

For both methoxychlor and *o*,*p*′-DDT, doses spaced about 10-fold apart produced essentially equivalent responses. This suggests either that the effect is not dose related or that the response from the doses tested is near the maximum for these pesticides. Because the lower doses produced a significant effect, a NOEL for acceleration of autoimmunity by these pesticides cannot be determined from this study. It is worthwhile noting that the lower dose of methoxychlor tested (3 mg/pellet, or approximately 1.2 mg/kg/day) is 4-fold lower than the NOEL used by the U.S. Environmental Protection Agency (EPA) in developing an oral reference dose for methoxychlor (U.S. EPA 2004). This suggests that an effect on autoimmunity might be a sensitive toxic end point (an effect that occurs at doses lower than other adverse effects) for methoxychlor, and therefore of particular interest for risk assessment. *o*,*p*′-DDT decreased the time to development of lupus at a dosing rate of 0.9 mg/pellet (or ~ 0.35 mg/kg/day). This is similar to the lowest observable effect level for *p*,*p*′-DDT (0.25 mg/kg/day) identified by the EPA based on liver changes in rats (U.S. EPA 2004), suggesting that autoimmune effects may be a sensitive end point for this OCP as well.

Changes in the time course for development of lupus produced by the OCPs in ovariectomized mice were comparable to that produced by 17β-estradiol, used in the study as a positive control for estrogenic effects. By and large, treatment of ovariectomized mice with the OCPs resulted in a rate of progression of disease that resembled its natural course in mice with intact estrogen, represented by the sham-surgery comparison group. These observations are consistent with an estrogenic mode of action for chlordecone, methoxychlor, and *o*,*p*′-DDT effects on autoimmunity. However, estrogenicity, as measured by uterine hypertrophy in the (NZB × NZW)F_1_ mice, correlated poorly with effectiveness in accelerating autoimmunity. Chlordecone had a greater effect on autoimmunity than either methoxychlor or *o*,*p*′-DDT but produced the same or less uterine hypertrophy at relevant doses. At lower doses where chlordecone was still effective in significantly decreasing the time to onset of renal disease, no significant effect on uterine weight was observed. The poor correlation might be due simply to differences in responsiveness to estrogenic effects by the uterus compared with the immune system. Unfortunately, there is little information in the literature that offers insight regarding this possibility.

Alternatively, the OCPs may influence autoimmunity through a mode of action unrelated to their estrogenicity. The potential importance of alternative actions is amply demonstrated by the recent study of Sawai et al. (2003), who fed female (NZB × NZW)F_1_ mice a diet containing bisphenol A. Bisphenol A is produced in the manufacture of plastics and has been shown to produce estrogen-like effects both *in vitro* and *in vivo*. In contrast to expectations based on estrogenicity, bisphenol A treatment significantly delayed the appearance of renal disease relative to controls. The authors attributed the delay by bisphenol A to an observed decrease in interferon-γproduction, an effect opposite of that known to be produced by estrogen.

In the experiments reported here we used a lupus model with a high genetic predisposition for disease. With this model, it was possible to demonstrate an effect of selected OCPs to modify the rate of progression of disease but not to test whether they are capable of influencing the incidence of disease. It remains to be determined whether these or other OCPs can initiate, on a susceptible genetic background, a break in tolerance—in other words, whether they can cause SLE in a susceptible individual that might not otherwise develop it. Answering this question will require testing OCPs in mouse strains with differing genetic background with respect to SLE susceptibility genes. Several such strains are available (e.g., Wakeland et al. 2001), and their use will be important in better characterizing the autoimmune hazard associated with OCP exposure.

## Correction

Values for body weight and dosing rate for chlordecone and the dosing rates for methoxychlor and *o,p*′-DDT were incorrect in the “Discussion” of the original manuscript published online. They have been corrected here.
